
JOURNAL INFORMATION
==============================
NLM Title Abbreviation: ScientificWorldJournal
Journal ID: ScientificWorldJournal
NLM Journal ID: 101131163
Journal ID: TSWJ

The Scientific World Journal

ISSN: 2356-6140
EISSN: 1537-744X
Publisher: Wiley

ARTICLE INFORMATION
==============================
PMCID: PMC5900861
PMID: 17704854
DOI: 10.1100/tsw.2007.231
Article ID: 534708
Article version: 1
Subjects: Directions in Science

Proteoglycan Biosynthesis during Chondrogenic Differentiation of Mesenchymal Stem Cells

Götting Christian *
Prante Christian
Kuhn Joachim
Kleesiek Knut
Institut für Laboratoriums- und Transfusionsmedizin, Herz- und Diabeteszentrum Nordrhein-Westfalen, Universitätsklinik der Ruhr-Universität Bochum, Bad Oeynhausen, Germany
*Christian Götting: cgoetting@hdz-nrw.deAcademic Editor: Martin Goette

Publication date: 2007
Electronic publication date: 2007 Aug 17
Volume: 7
First page: 1207
Last page: 1210
Received 2007 Jul 19; Revised 2007 Aug 1; Accepted 2007 Aug 6
Copyright: Copyright © 2007 Christian Götting et al.
Copyright year: 2007
License: This is an open access article distributed under the Creative Commons Attribution License, which permits unrestricted use, distribution, and reproduction in any medium, provided the original work is properly cited.
License URL: https://creativecommons.org/licenses/by/3.0/

Keywords: mesenchymal stem cells, chondrogenic differentiation, proteoglycan, glycosaminoglycan, xylosyltransferase, glycosyltransferase

Funding: Deutsche Vereinte Gesellschaft für Klinische Chemie und Laboratoriumsmedizin Ruhr-Universität Bochum F414-2004

==============================
Directions in Science
*TheScientificWorldJOURNAL* (2007) 7, 1207–1210
ISSN 1537-744X; DOI 10.1100/tsw.2007.231

### Proteoglycan Biosynthesis during Chondrogenic Differentiation of Mesenchymal Stem Cells

Christian Götting*, Christian Prante, Joachim Kuhn, and Knut Kleesiek

*Institut für Laboratoriums- und Transfusionsmedizin, Herz- und Diabeteszentrum Nordrhein-Westfalen, Universitätsklinik der Ruhr-Universität Bochum, Bad Oeynhausen, Germany*

**E-mail**: cgoetting@hdz-nrw.de

*Received July 19, 2007; Revised August 1, 2007; Accepted August 6, 2007; Published August 17, 2007*

**KEYWORDS**: mesenchymal stem cells, chondrogenic differentiation, proteoglycan, glycosaminoglycan, xylosyltransferase, glycosyltransferase

Adult mesenchymal stem cells (MSC) are multipotent progenitor cells that can be isolated from bone marrow, umbilical cord blood, or processed lipoaspirate[1], and that have the capability both to support hematopoiesis and to differentiate into at least adipocytes, chondrocytes, or osteoblasts[2]. Therefore, they represent suitable therapeutic tools for use in regenerative medicine and cell transplantation protocols[3]. MSCs are an attractive source of chrondrocyte precursor cells, which are of special interest for the *in vitro* generation of cartilage and its application in articular cartilage replacement therapy. While the capability of MSCs to differentiate into chondrocytes is well established, only limited knowledge about the formation and the remodeling of the cartilaginous extracellular matrix (ECM) during this process is available. The ECM is synthesized by the chondrocytes and is mainly composed of collagen fibrils, predominantly collagen type II, and proteoglycans, which form a matrix with high hydratative capacity. The biomechanical properties of cartilage are determined by a functional and intact ECM, and an imbalance of synthesis and degradation of matrix components leads to severe defects, like the loss of articular cartilage in osteoarthritis[4]. During chondrogenic stem cell differentiation, the biosynthesis and distinct deposition of proteoglycans is an important factor for the physiological efficiency of the cartilage, as the loading capacity in particular depends on the proteoglycan content in the cartilaginous matrix.

Proteoglycans are glycosylated macromolecules, which represent one of the most complex molecular structures in animal cells and which are essentially involved in many biological processes, including ECM deposition, biomechanical lubrication, cell-cell interactions, neurite outgrowth, or invasion strategies of pathogens[5]. They consist of a core protein to which one or more glycosaminoglycan chains are covalently attached[6]. The glycosaminoglycans chondroitin sulfate, dermatan sulfate, heparan sulfate, and heparin consist of disaccharide units that are attached to the core protein by a tetrasaccharide linker, glucuronic acid-β1,3-galactose-β1,3-galactose-β1,4-xylose-β-O-serine. The biosynthesis of the glycosaminoglycan chains occurs in the Golgi apparatus through the sequential addition of monosaccharides, which may finally be subjected to N- or O-sulfation, acetylation, deacetylation, or epimerization. These reactions are catalyzed by a distinct set of glycosyltransferases, epimerases, sulfotransferases, and other modifying enzymes[6,7]. The initial step in the post-translational biosynthesis

*Corresponding author.
©2007 with author.
Published by TheScientificWorld; www.thescientificworld.com of the uniform tetrasaccharide linkage region is catalyzed by the xylosyltransferases I and II (XT-I, XT-II, EC 2.4.2.26)[7,8,9,10]. These enzymes transfer a xylose residue from UDP-xylose to selected serine residues of the core protein and have been shown to catalyze the apparently rate-limiting step in glycosaminoglycan biosynthesis[7,11,12]. While being identified during a genetic screen for the XT-I cDNA sequences[13], the enzymatic activity of XT-II remained elusive until the end of 2006[10,14,15]. Both enzymes are capable of initiating chondroitin and heparan sulfate biosynthesis, and display some tissue-specific expression patterns[10,15]. Although the xylosylation of the core protein occurs in the early Golgi compartments, the xylosyltransferases are being secreted into the extracellular space together with proteoglycans[16,17]. Therefore, xylosyltransferase activity has been previously shown to be a reliable marker for the actual proteoglycan biosynthesis rate, which can be used for the assessment of tissue remodeling processes[16,18,19,20,21].

A successful and efficient biosynthesis of proteoglycans during the chondrogenic differentiation of MSCs requires the coordinated expression of both the core protein and multiple glycosyltransferases and modifying enzymes. Therefore, we have investigated the expression of key enzymes involved in the synthesis of chondroitin sulfate, dermatan sulfate, and heparan sulfate proteoglycans during TGF-$\beta_3$–induced chondrogenic differentiation of MSCs[22]. In order to differentiate between each pathway, expression of the key enzymes $\alpha$4-N-acetylhexosaminyltransferase (EXTL2), $\beta$1,4-N-acetylgalactosaminyltransferase (GalNAcT), and glucuronyl C5 epimerase (GlcAC5E) was analyzed. EXTL2 transfers N-acetylgalactosamine and N-acetylglucosamine to the uniform tetrasaccharide linker, and is a key enzyme for the synthesis of heparan sulfate. GalNAcT initiates the synthesis of chondroitin sulfate and dermatan sulfate glycosaminoglycans by catalyzing the transfer of the first N-acetylgalactosamine to the tetrasaccharide linker. The epimerization of chondroitin sulfate to dermatan sulfate is catalyzed by GalNAcT. The TGF-$\beta_3$–induced chondrogenic differentiation of MSCs resulted in the formation of cell aggregates that, after 28 days, showed a cartilage-like morphology. XT-I expression was up-regulated in early stages after TGF-$\beta_3$–induced chondrogenic differentiation, while XT-II was increased in the late stages. This is concordant with other findings from our group where XT-I, but not XT-II, expression was up-regulated in TGF-β–induced fibrotic processes[7,18]. It still has to be clarified whether this counterregulation of the xylosyltransferases leads to different proteoglycan patterns during chondrogenesis. Interestingly, all other glycosyltransferases showed a highly similar expression pattern, with a down-regulation in the early stages and a strong up-regulation after 1 week and more. The expression of major cartilage core proteins and type II collagen was considerably induced in the late stages of chondrogenic differentiation. All findings of the mRNA expression analysis could be paralleled with proteoglycan, glycosaminoglycan, or collagen content, as determined by enzyme activity, histochemistry, or immunostaining. The MSCs formed aggregates within 1 day after induction of chondrogenic differentiation and after 28 days, cryosections of the aggregates showed a condensed structure with chondrocyte-like lacunae after Safranin O staining, indicating the *in vitro* formation of cartilage.

The differentiation of MSCs into chondrocytes and the formation of intact cartilage with its important biomechanical properties require the coordinated biosynthesis of ECM components. The initiation of chondrogenesis leads to an induction of core proteins and glycosyltransferases involved in tetrasaccharide linker biosynthesis in the early stages of cell differentiation, while chain-elongating enzymes and type II collagen are rather up-regulated in later stages. These findings show a coordinated expression of genes involved in proteoglycan and collagen biosynthesis, although the underlying regulatory pathways have not been elucidated in detail. As proteoglycans have been shown to support collagen fibril formation in the ECM and both components are subject to cross-talk in the matrix network, the coordinated expression is supposed to be necessary for the formation of functional cartilage. While tissue-engineered cartilage has only limited biomechanical capacities compared to articular cartilage transplants, a modulation of these pathways might lead to an improved ECM formation and, consequently, overcome these limitations of *in vitro*–generated cartilage. This modulation of the proteoglycan biosynthesis pathway during the *in vitro* formation of articular cartilage could be achieved, e.g., by the addition of growth factors and cytokines, which are known as inducers of proteoglycan expression. Optimized cultivation and differentiation media containing these factors, e.g., from the TGF superfamily, would result in an enhanced expression of proteoglycans and other ECM molecules, leading to engineered cartilage with improved biomechanical properties and a broad range of therapeutic indications.

### ACKNOWLEDGMENTS

We thank Sarah L. Kirkby for her linguistic advice. This work was supported by the Stiftung für Pathobiochemie und Molekulare Diagnostik of the Deutsche Vereinte Gesellschaft für Klinische Chemie und Laboratoriumsmedizin and the Forschungsförderung an der Medizinischen Fakultät der Ruhr-Universität Bochum (FORUM), Grant F414-2004.

### REFERENCES

1. Bieback, K., Kern, S., Kluter, H., and Eichler, H. (2004) Critical parameters for the isolation of mesenchymal stem cells from umbilical cord blood. *Stem Cells* **22**, 625–634.
2. Pittenger, M.F., Mackay, A.M., Beck, S.C., Jaiswal, R.K., Douglas, R., Mosca, J.D., Moorman, M.A., Simonetti, D.W., Craig, S., and Marshak, D.R. (1999) Multilineage potential of adult human mesenchymal stem cells. *Science* **284,** 143–147.
3. Lee, J.W., Kim, Y.H., Kim, S.H., Han, S.H., and Hahn, S.B. (2004) Chondrogenic differentiation of mesenchymal stem cells and its clinical applications. *Yonsei Med. J.* **45,** 41–47.
4. Malemud, C.J. (1999) Fundamental pathways in osteoarthritis: an overview. *Front. Biosci.* **4,** D659–661.
5. Iozzo, R.V. (1998) Matrix proteoglycans: from molecular design to cellular function. *Annu. Rev. Biochem.* **67,** 609–652.
6. Kjellen, L. and Lindahl, U. (1991) Proteoglycans: structures and interactions. *Annu. Rev. Biochem.* **60,** 443–475.
7. Götting, C., Kuhn, J., and Kleesiek, K. (2007) Human xylosyltransferases in health and disease. *Cell. Mol. Life Sci.* **64,** 1498–1517.
8. Kuhn, J., Götting, C., Schnölzer, M., Kempf, T., Brinkmann, T., and Kleesiek, K. (2001) First isolation of human UDP-D-xylose: proteoglycan core protein beta-D-xylosyltransferase secreted from cultured JAR choriocarcinoma cells. *J. Biol. Chem.* **276,** 4940–4947.
9. Götting, C., Kuhn, J., Zahn, R., Brinkmann, T., and Kleesiek, K. (2000) Molecular cloning and expression of human UDP-d-Xylose:proteoglycan core protein beta-d-xylosyltransferase and its first isoform XT-II. *J. Mol. Biol.* **304,** 517–528.
10. Pönighaus, C., Ambrosius, M., Carrera Casanova, J., Prante, C., Kuhn, J., Esko, J.D., Kleesiek, K., and Götting, C. (2007) Human xylosyltransferase II is involved in the biosynthesis of the uniform tetrasaccharide linkage region in chondroitin sulfate and heparan sulfate proteoglycans. *J. Biol. Chem.* **282,** 5201–5206.
11. Seo, N.S., Hocking, A.M., Hook, M., and McQuillan, D.J. (2005) Decorin core protein secretion is regulated by N-linked oligosaccharide and glycosaminoglycan additions. *J. Biol. Chem.* **280,** 42774–42784.
12. Seo, N.S., McQuillan, D.J., and Hook, M. (2006) The role of glycosylation in the secretion of proteoglycans. *TheScientificWorldJOURNAL* **6,** 491–493.
13. Götting, C., Kuhn, J., Sollberg, S., Huerkamp, C., Brinkmann, T., Krieg, T., and Kleesiek, K. (2000) Elevated serum xylosyltransferase activity correlates with a high level of hyaluronate in patients with systemic sclerosis. *Acta Derm. Venereol.* **80,** 60–61.
14. Voglmeir, J., Voglauer, R., and Wilson, I.B. (2007) XT-II, the second isoform of human peptide-O-xylosyltransferase, displays enzymatic activity. *J. Biol. Chem.* **282,** 5984–5990.
15. Cuellar, K., Chuong, H., Hubbell, S.M., and Hinsdale, M.E. (2007) Biosynthesis of chondroitin and heparan sulfate in chinese hamster ovary cells depends on xylosyltransferase II. *J. Biol. Chem.* **282,** 5195–5200.
16. Götting, C., Sollberg, S., Kuhn, J., Weilke, C., Huerkamp, C., Brinkmann, T., Krieg, T., and Kleesiek, K. (1999) Serum xylosyltransferase: a new biochemical marker of the sclerotic process in systemic sclerosis. *J. Invest. Dermatol.* **112,** 919–924.
17. Schön, S., Prante, C., Bahr, C., Kuhn, J., Kleesiek, K., and Götting, C. (2006) Cloning and recombinant expression of active full-length xylosyltransferase I (XT-I) and characterization of subcellular localization of XT-I and XT-II. *J. Biol. Chem.* **281,** 14224–14231.
18. Prante, C., Milting, H., Kassner, A., Farr, M., Ambrosius, M., Schön, S., Seidler, D.G., Banayosy, A.E., Körfer, R., Kuhn, J., Kleesiek, K., and Götting, C. (2007) TGF-beta1 regulated xylosyltransferase I activity in human cardiac fibroblasts and its impact for myocardial remodeling. *J. Biol. Chem.*, in press.
19. Götting, C., Hendig, D., Adam, A., Schön, S., Schulz, V., Szliska, C., Kuhn, J., and Kleesiek, K. (2005) Elevated xylosyltransferase I activities in pseudoxanthoma elasticum (PXE) patients as a marker of stimulated proteoglycan biosynthesis. *J. Mol. Med*. **83,** 984–992.

20. Götting, C., Kuhn, J., Tinneberg, H.R., Brinkmann, T., and Kleesiek, K. (2002) High xylosyltransferase activities in human follicular fluid and cultured granulosa-lutein cells. *Mol. Hum. Reprod.* **8,** 1079–1086.
21. Schön, S., Huep, G., Prante, C., Müller, S., Christ, R., Hagena, F.W., Kuhn, J., Kleesiek, K., and Götting, C. (2006) Mutational and functional analyses of xylosyltransferases and their implication in osteoarthritis. *Osteoarthritis Cartilage* **14,** 442–448.
22. Prante, C., Bieback, K., Funke, C., Schön, S., Kern, S., Kuhn, J., Gastens, M., Kleesiek, K., and Götting, C. (2006) The formation of extracellular matrix during chondrogenic differentiation of mesenchymal stem cells correlates with increased levels of xylosyltransferase I. *Stem Cells* **24,** 2252–2261.

**This article should be cited as follows:**

Götting, C., Prante, C., Kuhn, J., and Kleesiek, K. (2007) Proteoglycan biosynthesis during chondrogenic differentiation of mesenchymal stem cells. *TheScientificWorldJOURNAL* **7**, 1207–1210. DOI 10.1100/tsw.2007.231.
